# High risk human papillomavirus and Epstein Barr virus in human breast milk

**DOI:** 10.1186/1756-0500-5-477

**Published:** 2012-09-01

**Authors:** Wendy K Glenn, Noel J Whitaker, James S Lawson

**Affiliations:** 1 School of Biotechnology and Biomolecular Sciences, University of New South Wales, Sydney, Australia

**Keywords:** Human papillomavirus, Epstein barr virus, Human milk

## Abstract

**Background:**

Multiple viruses, including human immunodeficiency virus, Epstein Barr virus (EBV) and mouse mammary tumour virus have been identified in human milk. High risk human papillomavirus (HPV) sequences have been identified in breast cancer. The aim of this study is to determine if viral sequences are present in human milk from normal lactating women.

**Findings:**

Standard (liquid) and *in situ* polymerase chain reaction (PCR) techniques were used to identify HPV and EBV in human milk samples from normal lactating Australian women who had no history of breast cancer.

High risk human papillomavirus was identified in milk samples of 6 of 40 (15%) from normal lactating women - sequencing on four samples showed three were HPV 16 and one was HPV 18. Epstein Barr virus was identified in fourteen samples (33%).

**Conclusion:**

The presence of high risk HPV and EBV in human milk suggests the possibility of milk transmission of these viruses. However, given the rarity of viral associated malignancies in young people, it is possible but unlikely, that such transmission is associated with breast or other cancers.

## 

High risk human papillomavirus (HPV) is primarily considered as a sexually transmitted infection [1]. However, a range of other means of transmission have been documented. These include white blood cells [2] oral mucosa [3] intrauterine, placenta and cord blood [4]. High risk HPV type 16 has been identified in 4 to 8% of human milk samples [5,6]. On the other hand, in a recent study Mammas et al [7] did not identify any high risk HPV sequences in human milk from Greek women. Other means of HPV transmission in addition to sexually transmitted infections are important factors to be considered in the context of primary prevention of HPV by vaccines. We have become aware of recent, but unpublished experimental findings, that Epstein Barr virus (EBV) has the capacity to enhance the oncogenicity of HPV associated cervical cancer cells [8]. For this reason, in addition to HPV, we also investigated the presence of EBV in human milk samples.

Here we report the identification of high risk HPV types 16 and 18 plus Epstein Barr virus (EBV) in milk from normal lactating Australian women. These findings have in part, been previously reported [9].

## Methods

This project was formally approved by the University of New South Wales, Australia, Human Research Ethics Committee (HREC 05163). All participants gave written, informed, prior consent.

Samples of human breast milk were collected from 40 lactating Australian women. None of these women had a history of breast cancer. The milk was expressed into sterile 50ml falcon tubes. To prevent contamination, the milk samples were then transferred into 1.5ml eppendorf tubes using filtered tips for all procedures. The 1.5ml tubes were centrifuged at 2,000 rpm for 10 minutes. The whey and fat were removed and the pellet was washed twice with phosphate buffered saline (PBS). The pellet was then resuspended in 200μl of PBS. 10μl 1M Tris pH8 and 10μl of 0.5M ethylenediaminetetraacetic acid (EDTA) were added to 100ul of the PBS based solution, the mixture vortexed and 2μl 2% sodium dodecyl sulfate (SDS) and 1μl proteinase K (10mg per ml) added. The tubes were heated at 55^o^C for 1-3 hours, after which a phenol extraction followed by 2 chloroform washes were performed before an alcohol precipitation. The DNA pellet was resuspended in Tris-EDTA (TE) and checked with a *β* -globin PCR analysis. A reagent blank of bovine skim milk was used for each set of milk extractions.

DNA integrity was confirmed by standard PCR using *β*-globin primers G073 (5′-GAAGAGCCAAGGACAGGTAC-3′) and G074 (5′-CAACTTCATCCACGTTCACC-3′). HPV screening used nested PCR with outer primers, PGMY, and inner primers GP as per Heng et al [10]. For EBV screening nested primers (EB3 5’-AAGGAGGGTGGTTTGGAAAG, EB4 5’-AGACAATGGACTCCCTTAGC) and (EB1 5’-ATCGTGGTCAAGGAGGTTCC, EB2 5’-ACTCAATGGTGTAAGACGAC) were used as per Cinque et al [11]. The cycling conditions for the above PCRs were 95°C for 3 min; followed by 35 cycles at 95°C for 30 seconds; 55°C for 30 seconds; 72°C for one minute and a final extension at 72°C for five minutes.

In situ PCR was attempted on a different set of milk samples. To prepare the milk samples for *in-situ* PCR, drops of the resuspended cell pellet in PBS were dried on glass slides followed by fixation in ice cold methanol for 5 minutes. An H&E stain of the slides identified the milk samples which contained cells. Selected samples were treated with 0.1% triton X digestion for 10 minutes at room temperature. The primers used for HPV *in situ* PCR were the outer primers used for the HPV screening. 75 μL of PCR mixture was prepared for each slide with the concentration of the reagents as follows: 7.5 μL 10X PCR buffer (Roche Diagnostics), 0.375 μL dNTP mixture (dATP, dGTP, dCTP; 25 mM/ml each), 0.33 μL digoxigenin (DIG) – 11 – dUTP (25 nM, Roche Diagnostics), 6 μL MgSO4 (100 mM, Roche Diagnostics), 1 μL (10pM) forward primer and 1 μL (10pM) reverse primer, 1 μL Taq Polymerase (Roche Diagnostics) and sterilized water. The thermocycling conditions were as follows: initial denaturation at 95°C for three minutes; 35 cycles at 95°C for one minute, 55°C for one minute and 72°C for one minute and a final extension at 72°C for five minutes.

## Findings

**Figure 1:**
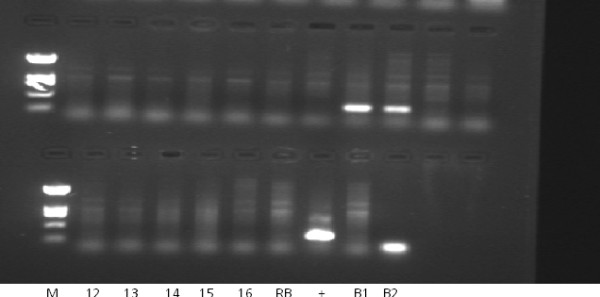
**Outcomes of standard liquid PCR using HPV primers.** 2% agarose gel of Nested PCR products from breast milk samples**.** M = Puc Hinf 1 size standard (1,419 bp, 517 bp plus 396 bp, 214 bp, 75/65 bp)**.** 1-40 = samples (only 1-16 shown)**.** RB = reagent blank**.** B1 = no DNA PCR blank after 2 round**.** B2 = no DNA PCR blank after 1 round**.** + = positive control (HeLa DNA).

**Figure 2:**
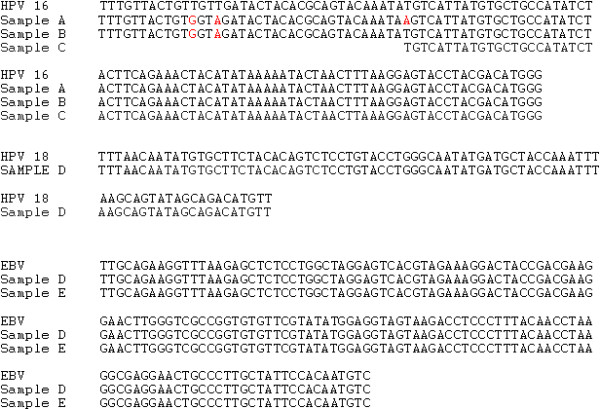
**Sequencing results.** Three HPV positive samples A,B,C compared to HPV 16 (FJ610151- bases 6627 to 6740) with differences shown in red, and one positive HPV sample D, compared to HPV 18 (AY262282). Samples C and D were only sequenced from one direction. Also sequences for two EBV positive samples D and E compared to V01555- (bases 109391- 109543) . Note that Sample D was positive for HPV and EBV. Primer sequences have been omitted.

Six of the forty milk samples (15%) were positive for HPV. Sequencing of four samples showed three were positive for HPV type 16 and one was positive for HPV 18 (Figures 1 and 2). Some variations in the HPV 16 sequences were present. While mutations at bases 6689 and 6715 are common, the mutation at base 6657, although reported before in AF548851 to be Y, has not been found to be an A. The HPV 18 sequence was identical to AY 262282.

Fourteen of the 40 milk samples (33%) were positive for EBV. Sequencing of two samples showed identical sequences to an EBNA 1 region of EBV strain B95-8. One of these samples was also positive for HPV 18.

**Figure 3:**
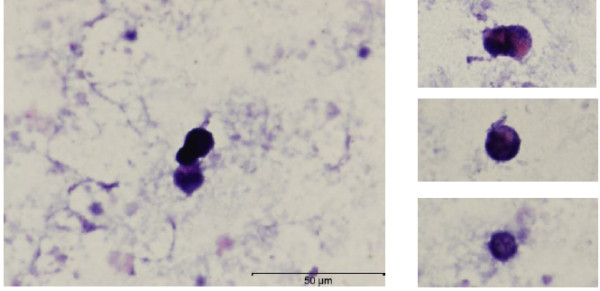
**HPV positive epithelial cell located in human milk by *in situ *PCR.** The images on the right have been cropped to show cells only. Some cells can be seen as groups. Dark blue colour indicates the incorporation of Dig dUTP in the PCR. The negative control with no primers added (not shown) had no blue colour in the cells.

*In-situ* PCR on a centrifuged milk sample showed high risk HPV in the nuclei of epithelial cells (Figure 3). There were differing amounts of cellular debris in each sample. Some appeared to contain no intact cells. The number of whole cells was very low and some were seen as clumps. The HPV positive cells seen in Figure 3 are from approximately 5% of the volume of a 1.5 ml milk pellet. There were approximately 10 HPV positive cells in the whole milk sample, no negative cells were observed. *In-situ* PCR was not performed on all the milk samples because of the low copy number of cells and large amounts of debris which were seen on the H&E. *In-situ* PCR for EBV was not performed.

## Discussion

We have demonstrated that both high risk HPVs and EBV may be present in the milk of a minority of normal lactating women, none of whom have had prior breast cancer. These findings are very similar to those of Sarkola et al [5] in Finland and Cazzaniga et al [6] in Italy and are in contrast to the negative findings of Mammas et al [7] in Greece.

Other viruses including human immunodeficiency virus (HIV), mouse mammary tumour virus and Epstein Barr virus have previously been identified in human milk [12-14]. Transmission of each of these viruses by human milk to newborn infants is a possibility. Mother-to-child transmission of HIV-1 infection during exclusive breastfeeding in the first 6 months of life is a common occurrence in Africa [12]. It is also known that HPV can be transmitted from infected mothers to their newborn babies with persistence of the virus into childhood [15]. It is not known whether HPV transmission can be from infected human milk.

High risk HPVs have been identified in both normal breast tissues and breast tumours of Australian women [10]. Therefore it is possible that HPV positive normal breast epithelial cells can be shed into the milk of normal lactating women.

The identification of viral sequences does not prove that these viruses are biologically active. The identification of HPV viral transcripts in human milk would be of future value. With respect to HPV such identification can indicate whether the HPV E6:L1 mRNA expression ratio is high (high E6 low L1) which is an indication that HPV is integrated and probably transforming, that is, the HPV is biologically active within the breast milk epithelial cells.

It is not known whether HPVs have long latency periods [16]. It is also unknown whether ingested HPVs can survive passage into the gut. HPVs are known to collaborate with Epstein Barr viruses which can have prolonged latency periods [8]. Therefore there are no current answers to the question whether HPVs and EBV ingested in infancy can lead to later oncogenicity.

## Abbreviations

EDTA: Ethylenediaminetetraacetic acid; H & E: Haematoxylin and eosin; HPV: Human papilloma virus; HIV: Human immunodeficiency virus; PBS: Phosphate buffered saline; PCR: Polymerase chain reaction; SDS: Sodium dodecyl sulfate; TE: Tris-EDTA.

## Competing interests

There are no conflicts of interest for any of the authors.

## Author’s contributions

WKG: collection of milk samples, conduct of standard and *in situ* PCR, preparation of the manuscript. NJW: supervision and quality assurance of the laboratory work, preparation of the manuscript. JSL: conceptualisation, collection of milk samples, analysis of data, manuscript preparation. All authors read and approved the final manuscript.
